# Processing, chemical signature and food industry applications of *Camellia sinensis* teas: An overview

**DOI:** 10.1016/j.fochx.2021.100160

**Published:** 2021-11-12

**Authors:** Débora Gonçalves Bortolini, Charles Windson Isidoro Haminiuk, Alessandra Cristina Pedro, Isabela de Andrade Arruda Fernandes, Giselle Maria Maciel

**Affiliations:** a Programa de Pós-Graduação em Engenharia de Alimentos (PPGEAL), Universidade Federal do Paraná (UFPR), CEP (81531-980) Curitiba, Paraná, Brazil; b Laboratório de Biotecnologia, Universidade Tecnológica Federal do Paraná (UTFPR), CEP (81280-340) Curitiba, Paraná, Brazil

**Keywords:** *Camellia sinensis*, Withering, Fermentation, Theaflavin, Catechin, Caffeine

## Abstract

The plant *Camellia sinensis* is the source of different teas (white, green, yellow, oolong, black, and pu-ehr) consumed worldwide, which are classified by the oxidation degree of their bioactive compounds. The sensory (taste, aroma, and body of the drink) and functional properties of teas are affected by the amount of methylxanthines (caffeine and theobromine), amino acids (l-theanine) and reducing sugars in their composition. Additionally, flavan-3-ols, mainly characterized by epicatechins, catechins, and their derivatives, represent on average, 60% of the bioactive compounds in teas. These secondary metabolites from teas are widely recognized for their antioxidant, anti-cancer, and anti-inflammatory properties. Thus, *Camellia sinensis* extracts and their isolated compounds have been increasingly used by the food industry. However, bioactive compounds are very susceptible to the oxidation caused by processing and degradation under physiological conditions of gastrointestinal digestion. In this context, new approaches/technologies have been developed for the preservation of these compounds. This review presents the main stages involved in production of *Camellia sinensis* teas following a description of their main bioactive compounds, biological properties, stability and bioaccessibility. Besides, and updated view of *Camellia sinensis* teas in the field of food science and technology was provided by focusing on novel findings and innovations published in scientific literature over the last five years.

## Highlights

• The oxidation degree of phenolic compounds (PC) results in different types of teas.
• Flavan-3-ols, 60% of PC in teas, highly contribute to biological activities.
• New methods for teas manufacturing are required to ensure higher stability of PC.
• Phenolic compounds from teas are also unstable to gastrointestinal digestion.
• Biosorption and encapsulation of PC are alternatives for their preservation.

## Introduction

Teas are the second most consumed beverage after water (Paiva, Lima, Motta, & Marcone, 2020). The term tea is assigned exclusively to non-alcoholic caffeinated beverages obtained by the infusion process of the *Camellia sinensis* plant from China. Therefore, the hot drink obtained from other plants is simply called an infusion (Shang, Li, Zhou, Gan, & Li, 2021). However, teas are commonly prepared as an infusion technique, which consists of adding the plant matrix in a container with boiling water (up to 100 °C), capping, and letting it rest from 5 to 10 min. Subsequently, the liquid is used for consumption or scientific research (Chávez-González et al., 2020).

*Camellia sinensis* flowers and leaves have a rich composition of bioactive compounds such as phenolic compounds (phenolic acids, flavonoids and tannins), alkaloids (methylxanthines) and nutrients (carbohydrates, proteins and minerals) (Sharma, Verma, & Kumar, 2021). The chemical composition of the flower, from which white tea originates, consists of 34% carbohydrates (glucose, fructose, sucrose, and polysaccharides), 12% phenolic compounds (PCs), 28% crude proteins and 3% saponins. Additionally, it contains a wide variety of amino acids, such as aspartic acid, serine, histidine, arginine, γ-aminobutyric acid, threonine, tyrosine, valine, methionine, leucine, phenylalanine, lysine and theanine (Chen, Ding, et al., 2020). The composition of dry leaves has from 1 to 4% of amino acids (26 amino acids already reported in the literature) and 18 to 36% of phenolic compounds (PCs), including flavonols, flavonoids and phenolic acids. From 12 to 24% of these PCs are catechins (Chen, Wang, Tsai, et al., 2021). The phenolic composition of *C. sinensis* is responsible for its antioxidant activity. This property plays in important role to human health, as it inhibits or reduces the effects of aging and chronic-degenerative diseases (Shang et al., 2021). Other biological properties from different bioactive compounds from teas such as, modulating bowel health, improving induced immunosuppression (Chen et al., 2019, Chen et al., 2020) and reducing obesity in rats (Sun, Xu, Ye, & Gaikwad, 2019) were also reported.

However, bioactive compounds profile may be modified during tea processing since the exposition of oxygen can imply on degradation of some chemical compounds. Thus, teas are classified according to their processing, where the main variation occurs in the degree of oxidation that modifies chemical and sensory characteristics (Chen, Liu, et al., 2021).

In this context, this review addresses the growing number of studies and research areas related to *C. sinensis* over the past five years. The review article also presents an overview of the effect of processing on bioactive compounds, the main aspects related to the bioaccessibility of bioactive compounds from *C. sinensis*, viable alternatives for the preservation of these compounds and how their biological actions stand out in the food industry segment. Thus, this review was prepared with recent studies, primarily from 2019, totaling 98 scientific papers. The databases used for the research included Science Direct and Google Scholar, as well as statistical data on production and recent studies made available by Web of Science and Food and Agriculture Organization (FAO). The main keywords used in the searches were *Camellia sinensis*, tea, withering, tea fermentation, yellowness, tea health promotion and tea technology.

## Camellia sinensis

**Fig. 1:**
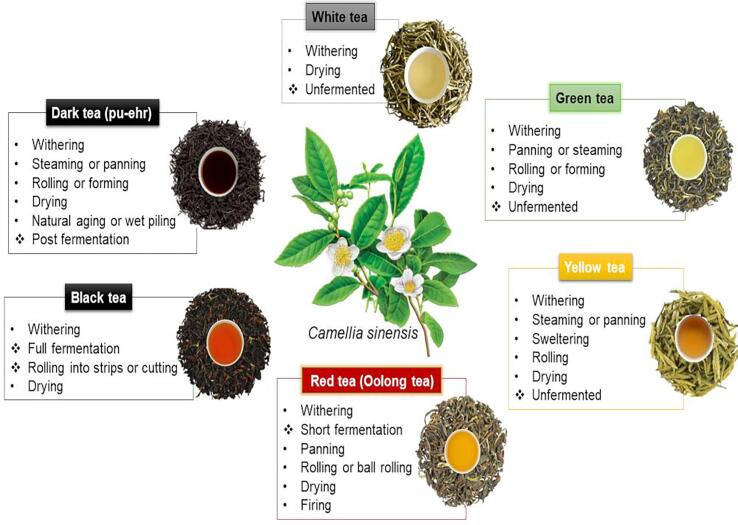
Types of tea (*C. sinensis*) according to processing.

*Camellia sinensis*, native to China and Southeast Asia, has spread around the world primarily as a traditional medicine. This plant is distinguished according to its morphological characteristics and can be divided into *C. sinensis* var. *sinensis* (shrub with small or medium leaves) and *C. sinensis* var. *assamica* (tree with large leaves) (Jia, Zhang, Fernie, & Wen, 2021). The chemical composition, types, qualities and characteristics of flavor, aroma and color of tea are mainly associated with processing (harvesting, cleaning the leaves, selection, partial or total drying of the leaves, rolling and storage), as well as the degree of fermentation (unfermented, partially fermented, fully fermented and post-fermented) (Chen et al., 2021, Shang et al., 2021). In this way, tea can be classified (Fig. 1) into white tea, green tea, yellow tea, oolong tea (also known as red tea), black tea and dark tea (pu-erh) (Chen, Wang, Tsai, et al., 2021).

In 2019, the production of tea for consumption and research was around 5.79 million tons (corresponding to an area of 1 million hectares), generating revenue of US$ 7 billion dollars in world export (FAOSTAT, 2021). Countries like China and India concentrate 72% of this production, earning a combined revenue of approximately US$ 3 billion in exports.

**Fig. 2:**
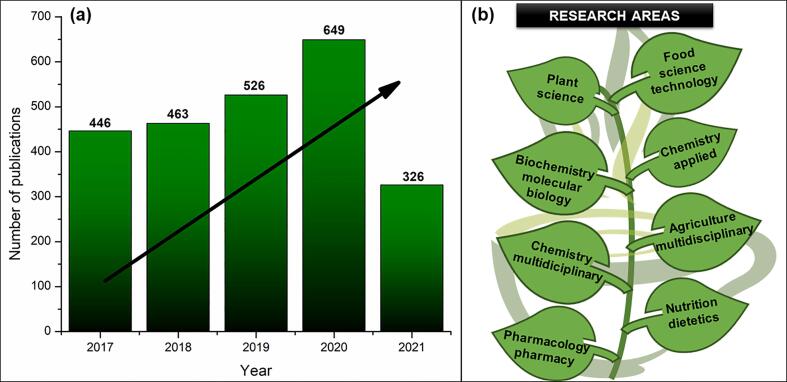
(a) Articles on *Camellia sinensis* published in journals between 2017 and 2021 (August 4th, 2021); (b) Main areas of study on *C. sinensis.*

Studies on *C. sinensis*, ranging from the improvement of extraction process conditions to the analysis of possible applications in different areas of research, have grown substantially in recent years. The number of indexed articles that studied *C. sinensis* and were published in scientific journals from 2017 to 2021 increased in this period (>46%), corresponding to a total of 2410 publications in 5 years (until August 4, 2021) (Fig. 2a). Different fields of research, such as food science, plant science, applied chemistry, molecular biology, biochemistry, among others have developed studies involving *C. sinensis* (Fig. 2b). The main topics investigated are related to the bioactive composition, antioxidant properties, biological activities and the use extracts or isolated compounds of teas to develop new products of interest to food and pharmaceutical industries (Web of Science Database, 2021).

Over the years, teas became more than simply pleasing and cultural hot drinks. Teas have prevailed for their hypoglycemic, antioxidant, anti-inflammatory, neuroprotective, anticarcinogenic, anti-obesity, cardiovascular and liver protection properties, especially when consumed through functional foods or food supplements (Shang et al., 2021). These biological activities are mainly associated with teas richness in bioactive compounds, such as phenolic acids and flavonoids (Chen, Wang, Tsai, et al., 2021).

## Bioactive composition of teas

The chemical composition of tea, especially phenolic compounds, can vary according to geographic, genetic, ecological, physiological and processing factors (Abdel Azeem et al., 2020, Pedro et al., 2019). Processing plays an important role in the phenolic composition, since some compounds are evidenced only after the fermentation of the leaves (Abdel Azeem et al., 2020), as well as some compounds are degraded during this process (Chen, Liu, et al., 2021). An example of this are the higher levels of theanine contained in green tea (unfermented) in comparison with black tea (fully fermented) (Abdel Azeem et al., 2020). Likewise, the highest concentration of theaflavin (obtained in the process of oxidizing the leaves), the darker the tea (Braibante, da Silva, Braibante, Hugo, & Pazinato, 2014).

Scientific studies on teas have been carried out using various analytical techniques of analysis (colorimetric, high-performance liquid chromatography and mass spectrometry) for the determination of bioactive composition (Chen et al., 2021, Chen et al., 2021). Among the compounds present in teas (Table 1), amino acids, methylxanthines (caffeine and theobromine) and theanine (which represents 60–70% of the total amino acid in tea leaves) stand out, as well as phenolic compounds such as phenolic acids, catechins and flavonoids (Fang et al., 2017). However, it is important to note that the variation in the concentration of bioactive compounds is influenced by the different stages of tea processing.

**Table 1:** Bioactive compounds in teas.

Bioactive compounds (mg/g)	*Camellia sinensis*					
	White tea	Green tea	Yellow tea	Oolong tea	Black tea	Dark tea
*Phenolic acids*						
Gallic acid	2.09–2.71^(1)^	0.64–2.97^(9)^	1.97–3.16^(1,13)^	0.29–3.05^(1)^	2.21–6.37^(9)^	13.38–18.06^(14)^
Ellagic acid	2.30–2.35^(1)^	2.33–7.77^(1)^	2.39–9.79^(1)^	2–10-2.29^(1)^	2.11–4.57^(1)^	2.31–2.72^(1)^
Chlorogenic acid	ND^(1)^	0.07–0.51^(9)^	0.26–0.35^(1)^	0.24–0.30^(1)^	0.18–0.41^(9)^	0.35^(1)^
Ferulic acid	–	3.33–7.14^(9)^	–	–	4.74–8.20^(9)^	–

*Cathechins*						
(+)-catechin	1.02–6.23^(2,3)^	1.13–6.42^(1,3,10)^	0–95-1.40^(1,13)^	0.85 ^(2)^	0.33^(2)^	ND^(1)^
(−)-epicatechin	0.83–11.16^(1,3)^	3.18–14.21^(3,11)^	1.96–3.03^(13)^	2.45–9.19^(1)^	0.45–1.33^(1)^	1.10–8.28^(15)^
(−)-epigallocatechin	6.69–14.71^(1,3)^	6.21–21.02^(3,11)^	6.18–6.52^(13)^	16.22–73.97^(1)^	0.91^(2)^	2.90–16.76^(15)^
(−)-epigallocatechin gallate	2.11–50.3^(1)^	90–64-95.50^(11)^	35.59–52.23^(13)^	10.34–34.74^(1)^	1.34–2.15^(1)^	16.95–22.51^(15)^
(−)-epicatechin gallate	2.67–14.32^(1)^	6.71–22.64^(11)^	8.10–13.20^(13)^	3.08–7.57^(1)^	1.12–2.62^(1)^	61.17^(15)^
Total catechins	**78.15**–**86.07**^(3,4)^	**106.02–145.54**^(10,11,12)^	**67.36**–**82.78**^(13)^	**70.31–136.88**^(12)^	**3.95–38.69**^(12)^	**20.95**–**152.63**^(14,15)^
Total flavonoids	**28.30**–**33.7**^(4,5)^	**1.16**–**29.9**^(10,11)^	**17.30**^(7)^	**15.07**^(7)^	**6.36**–**62.10**^(3,8)^	**3.02–20.15**^(7,14,15)^
Total phenolics compounds	**32.53**–**75.7**^(4,6)^	**12.36**–**252.65**^(1,6,10,11)^	**39.55**–**220.08**^(1,6)^	**31.33**–**150.10**^(1,6)^	**11.33**–**101.29**^(1,4,6)^	**11.95–147.11**^(1,6)^

*Amino acids*						
l-Theanine	7.53–11.91^(3,7)^	2.63–14.23^(3,12)^	9.49^(7)^	1.70–8.38^((3,12)^	1.43–11.00^(3,7)^	1.83–11.49^(7,15)^
γ-aminobutyric acid	0.11–1.67^(3,7)^	0.02–1.12^(3,7)^	0.27^(7)^	0.35^(7)^	0.06–0.79^(3,7)^	0.03^(7)^
Total free amino acids	**29.64**^(7)^	**26.05**^(7)^	**21.59**^(7)^	**21.85**^(7)^	**13.38**^(7)^	**4.29**^(7)^

*Metilxanthines*						
Theobromine	0.40^(2)^	3.95–8.39^(9)^	0.40^(13)^	0.28^(2)^	0.70–4.43^(9)^	1.84–15.57^(14,15)^
Caffeine	19.28–27.54^(8)^	2.64–42.20^(8,9)^	24.49–39^(8,13)^	12.36–31.66^(8)^	4.05–39.55^(8,9)^	8.94–125.86^(14,15)^

**Note:** ND: not detected. ^(^*^n^*^)^References by column: ^(1)^Zhao et al. (2019); ^(2)^Sun et al. (2019); ^(3)^Li, Zeng, Liao, Tang, and Yang (2020); ^(4)^Ribeiro et al. (2021); ^(5)^Liu et al. (2019); ^(6)^Klepacka, Tońska, Rafałowski, Czarnowska-Kujawska, and Opara (2021); ^(7)^Jiang et al. (2019); ^(8)^Samadi and Raouf Fard (2020); ^(9)^Azevedo et al. (2019); ^(10)^Tang et al. (2021); ^(11)^Liu et al. (2020); ^(12)^Chen, Wang, Tsai, et al. (2021); ^(13)^Zhou et al. (2019); ^(14)^Armstrong et al. (2020); ^(15)^Zhou, Ma, Wu, Xu, & Wang (2020).

Catechins are colorless compounds insoluble in water, which are responsible for the bitterness and astringency of teas. The variation in catechin concentrations in each type of tea is responsible for sensory attributes (aroma, color and flavor) and different biological activities, such as reducing premature aging, protective effects on the neurological and cardiac systems, and anticholesterolemic, antitumor and anti-inflammatory properties (Pedro et al., 2019). In addition, the flavan-3-ols in teas are capable of inhibiting the activity of the main protease (M^pro^) of SARS-Cov-2 (Zhu & Xie, 2020). Also, catechins show a high degree of oxidation during processing. Thus, the less processing to which *C. sinensis* is submitted, the higher the concentration of catechins (Braibante et al., 2014).

The high concentration of different PCs present in teas is responsible for its antioxidant activity. These antioxidants act as a neutralizing agent for free radicals that cause oxidative stress, inhibiting or decreasing the effects of aging and chronic-degenerative diseases (Shang et al., 2021). However, PCs, especially phenolic acids, are unstable and easily oxidized. Thus, it should be considered that the increase in oxygen concentrations during the tea fermentation process can promote the oxidation of catechins, glycosylated flavonoids and some phenolic acids. Furthermore, reduced concentrations of secondary metabolites are also associated with amino acid degradation (to form volatile aldehydes). This degradation is the result of the oxidation reaction of catechins that consequently promote the oxidation of some phenolic acids and the reduction of astringency in flavor attributes (Chen, Liu, et al., 2021).

In addition to phenolic compounds, *C. sinensis* contains in its composition secondary metabolites derived from purine nucleotides, in particular, methylxanthines that are soluble in water and have a high capacity to stimulate the central nervous system (Zhang et al., 2019). Among the methylxanthines in tea, there is caffeine in higher concentrations and theobromine, which are mainly responsible for the quality of the tea (Teng et al., 2020). Caffeine stands out for being a fat-soluble substance that has a stimulating effect, promoting a condition of more attention and concentration, enhancing fat burning (when associated with exercise) and also reducing mental fatigue (Chirasani, Pasek, & Meissner, 2021).

The identification and study of the interaction mechanisms of bioactive compounds are as important as the quantification of concentration levels. Liao et al. (2021) studied protein–protein, chemical-protein, and chemical-chemical interactions of *C. sinensis* teas. Among the 47 compounds identified are cinnamic acid, p-coumaric acid, catechin, cyanidin, delphinidin, epigallocatechin (EGC), epicatechin gallate (ECG), epigallocatechin gallate (EGCG), gallocatechin (GC), phenylalanine, myricetin and quercetin. In another study, Tang, Guo, Zhang, Yang, and Song (2021) identified through the high-performance thin-layer chromatography technique (HPTLC) 44 phenolic compounds, among phenolic acids, catechins, tannins and flavonols in tea extract.

This phenolic composition of tea is an important antioxidant protection factor obtained from food sources, as these antioxidants reduce cell damage caused by free radicals through chelating processes with transition metals, maintaining the biological functions of DNA, proteins and lipid membranes (Shang et al., 2021). Particularly in tea consumption, the temperature (up to 95 °C) of the aqueous infusion process responsible for extracting the phenolic compounds from the matrix, does not degrade these compounds. However, the digestive process impacts the degradation of PCs and the antioxidant potential of teas; therefore, it is necessary to develop phenolic delivery systems that guarantee the protection of bioactivity during digestion and promote better absorption of these compounds by the intestinal microbiota (Jilani, Cilla, Barberá, & Hamdi, 2020).

### Stability of bioactive compounds from teas

Bioactive compounds are sensitive to oxidation, hydrolysis, and other chemical reactions. These reactions occur during processing, where part of the compounds are oxidized (Paiva et al., 2021, Rahman et al., 2020), and after drinking the infusion, where gastrointestinal digestion can change chemical structures, resulting in compounds with different bioactivities (Jilani et al., 2015, Ribeiro et al., 2021). In order to increase the stability of phenolic compounds and take advantage of their bioactive properties, technologies have been developed for several different industrial areas (Jilani et al., 2020, Ribeiro et al., 2019, Silva et al., 2021). This topic deepens the study of the stability of bioactive compounds in teas during processing and after the gastrointestinal digestion of infusions.

#### Effect of tea processing on bioactive content of teas

**Fig. 3:**
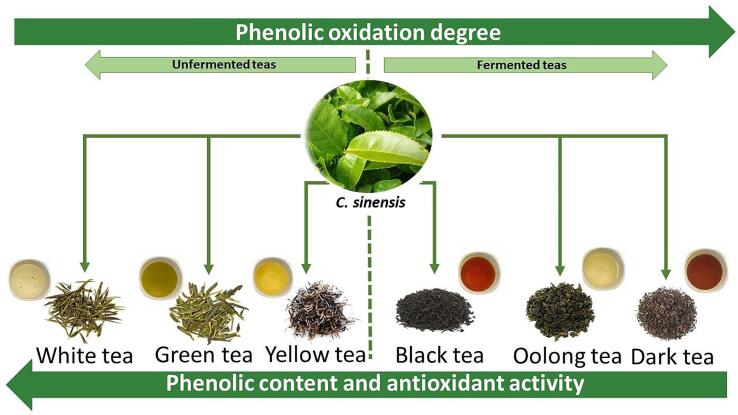
Effect of tea processing on phenolic content and antioxidant activity of infusions.

The unit operations involved in tea processing (Fig. 1) directly influence its chemical composition (Table 1) (Sun et al., 2019), impacting its bioactive properties, such as antioxidant and sensory activity. The production of tea herbs involves up to five-unit operations, known as withering, panning or steaming, sweltering, rolling, and drying. The more steps, the higher the reduction in the concentration of bioactive compounds (Braibante et al., 2014), mainly phenolic compounds. Furthermore, these operations alter the concentration of methylxanthines, theaflavins, amino acids and volatile compounds, and consequently their bioactive properties, resulting in different types of teas (Fig. 3). As can be seen in Table 1, each type of *C. sinensis* tea, which goes through different processing stages, has different concentrations of phenolic compounds.

The withering step aims to reduce the moisture in the tea leaves, making them soft and leathery (Rahman et al., 2020) and to modify sensory characteristics of teas such as taste and aroma. During this step, the phenolic profile is modified, especially in flavan-3-ols, predominantly epicatechins (90%), which represent between 60 and 70% of the total phenolic compounds (Paiva et al., 2021). These flavonoids are mainly responsible for the bitterness and astringency of teas (Fang et al., 2017). There is also the formation of volatile compounds, responsible for the aroma of teas. Chen, Zhu, et al. (2019) found 172 different volatile compounds, which were formed and/or modified during the withering step in white tea. Withering reduces the concentration of reducing sugars and increases the concentration of caffeine in tea, as well as oxidase enzymes. The polyphenol oxidase (PPO) and peroxidase (POD) enzymes play an important role in tea processing since catalyze the oxidation reactions of phenolic compounds (Rahman et al., 2020). Yılmaz, Özdemir, and Gökmen (2020) demonstrated that the total free amino acid content of teas increased in the withering stage while decreasing in the drying stage, proving that different stages of tea processing affect the level of GABA (Gamma-aminobutyric acid), kynurenic acid, and dopamine. According to these authors, GABA and kynurenic acid concentration of tea increased 33% and 53% after withering, respectively. In comparison, the tea that went through the drying process showed lower GABA and kynurenic acid than withered tea.

Among the studied teas, white tea presents a higher concentration of phenolic compounds, due to the simpler processing, which involves only the withering steps, followed by drying (Fig. 1). In this way, the process of obtaining white tea dispenses with any steps of enzymatic inactivation and fermentation, providing an unequaled flavor. Their flavor is described as slightly sweet/umami and fresh/green odor (Chen, Zhu, et al., 2019). Therefore, white tea is considered an unfermented tea with a low degree of oxidation of phenolic compounds (Paiva et al., 2021).

The steaming step, also known as panning or roasting, is carried out in order to inhibit enzymes responsible for the enzymatic browning process (Donlao & Ogawa, 2019). In the presence of oxygen, the enzyme group of polyphenol oxidase (PPO), catalyzes the hydroxylation of monophenols, forming *o*-diphenols, followed by the oxidation of *o*-diphenols, resulting in *o*-quinones and dark pigments called melanoidins (Zhou et al., 2020). As these enzymes are thermosensitive, the steam treatment is effective in enzymatic denaturation (Donlao & Ogawa, 2019). However, phenolic compounds are also susceptible to degradation at high temperatures. Thus, the time and temperature used in the process must be strictly controlled during the process. High pressure assisted freezing (Cheng, Zhu, & Sun, 2021) and high-intensity ultrasound (Habuš et al., 2021) may be alternatives for the denaturation of PPO, reducing damage to the concentration of polyphenols. The residual content of this oxidase in teas can also be inhibited by ultra-high pressure (Chen et al., 2021, Chen et al., 2021).

Green tea, an unfermented product, has a loss of around 30% of catechins that are oxidized in the withering process. Green leaves go through an enzymatic inactivation step, where the polyphenol oxidase (PPO) is inactivated by steam (steaming or roasting), which occurs between 200 and 300 °C, reducing the amount of flavan-3-ols. In addition, drying, which can take place between 80 and 160 °C, can also reduce the concentration of catechins by thermal degradation in the final product (Donlao & Ogawa, 2019). Processing green tea can also change the color of dry leaves due to changes in chlorophyll. However, the modification of the concentrations of these compounds does not influence the color of the infusion (Donlao & Ogawa, 2019).

The processing of yellow tea differs from white and green teas due to the presence of an additional processing step, which is known as sweltering or yellowing (Fig. 1). At this stage there is a decomposition of chlorophyll, a reduction in the level of amino acids and flavan-3-ols, such as epigallocatechin gallate (EGCG), epigallocatechin (ECG), gallocatechin (GC) and procyanidin B2. There is also an increase in the levels of pipecolic acid, betaine, lysophosphatidylcholines and theasinensin B. Therefore, this combination of factors and metabolites is responsible for the significant changes in the color and flavor of this tea (Wei et al., 2021).

Leaf size reduction in the rolling step is performed for all types of tea, except for white tea (Fig. 1). This reduction increases the surface in contact with oxygen. Fermented teas do not undergo steaming prior to fermentation. Thus, during the rolling, the PPOs and PODs, coming from the chloroplasts and the plant cell wall, respectively, are exposed to the medium (Salman, Yılmaz, Gökmen, & Özdemir, 2021).

The fermentation process represents the main step in the manufacturing for some teas. Fermentation, in this case, refers to an enzyme-catalyzed oxidative process, without the involvement of microbiological agents (Salman et al., 2021). At this stage, important biochemical changes occur, such as the oxidation of phenolic compounds, resulting in the appearance of theaflavins and thearubigins (Rahman et al., 2020). Theaflavins, formed by the condensation of orthoquinones from the oxidation of catechins, are responsible for the beverage's brightness and liveliness. On the other hand, thearubigins, a product of the oxidation of gallic acids, which form polymerized epitheaflavic acids, characterize the infusion's color, flavor and body (Rahman et al., 2020). Fermented teas differ in fermentation time and can be classified as short fermentation (oolong tea), full fermentation (black tea) and fermented after processing (pu-ehr tea) (Fig. 3).

Drying is carried out as the final step in the processing of teas, aiming at removing water, which improves its storage and transportation. Another purpose of drying is the denaturation of oxidases after the oxidation process of some teas (Salman et al., 2021). Conventional drying is carried out by the application of hot air (Shi et al., 2019). As with other thermal processes, drying can reduce the concentration of bioactive compounds in dry matter, and technologies have been developed to preserve these chemical compounds. Drying technologies, such as the use of microwave, far infrared, halogen lamps, and halogen-microwave lamps were compared with the conventional (hot air) tea drying method (Qu et al., 2019). The concentrations of phenolic compounds, theaflavins, amino acids, soluble sugars and volatile compounds in dry black tea were evaluated using the different technologies. Drying by microwave and halogen-microwave lamp showed the best results in the preservation of the analyzed compounds (Qu et al., 2019).

As already discussed, the preservation of bioactive compounds in the leaves of *C. sinensis* is correlated with the control of the processing steps. In addition to the compounds instability during processing, after ingestion of beverages, these compounds undergo different chemical reactions, capable of transforming them into different compounds, increasing or reducing their bioactive potential (Jilani et al., 2020).

#### Bioaccessibility of bioactive compounds from teas

Gastrointestinal digestion aims to reduce the size of macronutrients, such as breaking down proteins into amino acids, hydrolysis of some sugars into monosaccharides, obtaining free fatty acids from extensive lipid molecules, in addition to releasing micronutrients present in the solid food medium. These physiological modifications enable a better absorption of these nutrients. However, food matrices contain compounds sensitive to physiological conditions, such as changes in pH and the presence of enzymes and bile salts, present in the gastrointestinal environment. The exposure of bioactive compounds to this environment can change its chemical structure, resulting in new compounds, with or without biological properties (Jilani et al., 2015).

The bioaccessible fraction of these compounds refers to their concentration remaining after gastrointestinal digestion, being available for intestinal absorption. Thus, bioaccessibility is defined as the amount of bioactive compounds available for absorption after gastrointestinal digestion (Jilani et al., 2020), whereas bioavailability refers to the content of bioactive compounds found in blood and/or tissues after intestinal absorption (Cai et al., 2018).

Rusak, Šola, and Vujčić Bok (2021) evaluated the effect of simulated gastrointestinal digestion on the concentration of phenolic compounds, antioxidant activity, and antidiabetic potential of powdered green tea Matcha and bagged Sencha. Sencha green tea is obtained from conventional manufacturing while the production of Matcha tea involves the inhibition of approximately 90% of sunlight between 3 and 4 weeks before harvest, accelerating the maturation of the leaves. Aqueous extracts (10 g/L, diluted in water at 80 °C) were obtained and subjected to simulated salivary, gastric, and intestinal digestion. The total concentration of phenolic compounds reduced from 68% to 79%, of which flavan-3-ols reduced between 83 and 88% and flavonols, 51 to 58% after simulated gastrointestinal digestion. After the digestive process, the antidiabetic activity of Matcha tea was superior to Sencha, standing out as an adjunct in the treatment of postprandial hyperglycemia (Rusak et al., 2021). Shu, Li, Yang, Dong, and Wang (2019) evaluated the bioaccessibility of phenolic compounds from powdered green tea sieved in different particle sizes (564.24, 47.85, 34.62 and 15.10 µM). After simulated gastric digestion, bioaccessibility ranged from 59.98 to 71%, and after simulated intestinal digestion these values reduced from 9.69 to 15.57%. In this study, it was found that reducing the size of powdered green tea particles to 34.62 µM has higher antioxidant activity after simulated gastrointestinal digestion.

Due to the instability of phenolics under physiological conditions, technologies have been improved/developed for the preservation of such compounds during gastrointestinal digestion. Microencapsulation has been used as a method to protect phenolic compounds to increase their bioaccessibility. The microencapsulation of these compounds in cashew gum and maltodextrin, as well as the gastroprotective function of these capsules were studied (Silva et al., 2021). Microencapsulation increased the bioaccessibility of phenolic compounds in green tea by 28.2% and increased the antioxidant activity by 24.2% after simulated gastrointestinal digestion. The concentration of 10 mg/kg of microcapsules containing green tea and green tea extract had a gastroprotective effect in rats (Swiss mice male, n = 18) due to the maintenance of glutathione in lesions induced by absolute alcohol (Silva et al., 2021).

The biosorption of phenolic compounds presents a viable alternative for the preservation of bioactive compounds under physiological conditions of gastrointestinal digestion. The biosorption of phenolic compounds uses by-products from food industry, such as *Saccharomyces cerevisiae* yeasts from beer production. Yeasts are recovered from production, washed until any brewing residue is exhausted and dried or freeze-dried. Afterwards, acid and alkaline treatments are carried out for in order to improve the adsorption capacity of the biosorbent (Ribeiro et al., 2019). Jilani et al. (2015) were pioneers in the study of biosorption of phenolic compounds from green and black teas in *Saccharomyces cerevisiae* yeasts. In this study, the biosorption process was able to increase by 10.91% and 13.19% the bioaccessibility of total phenolic compounds for black tea and green tea, respectively. For the TEAC antioxidant activity, this increase was from 11.07% (black tea) to 32.46% (green tea), and the bioaccessibility of the antioxidant activity by the ORAC method increased around 47% after biosorption for both the studied teas. The same research group evaluated the antiproliferative effect of of green and black teas polyphenols on Caco-2 colon cancer cells after biosorption and simulated gastrointestinal digestion. The bioaccessible extracts did not show cytotoxicity, and a reversible arrest of the cell cycle without induction of apoptosis was observed, which indicates cytostatic activity of these digested infusions (Jilani et al., 2020).

Our research group has also been investigating the preservation of phenolic compounds via biosorption (delivery system model) in *S. cerevisiae* yeast (Oliveira et al., 2019, Rossetto et al., 2020, Rubio et al., 2018, Stafussa et al., 2016). Ribeiro et al. (2021) observed that *S. cerevisiae* cells were able to biosorb more than 50% of the total phenolic compounds from white tea and green tea. After simulated gastrointestinal digestion, the bioaccessibility of total phenolic compounds in tea extracts ranged between 12.2% (green tea) and 18.8% (white tea), while in yeasts, the bioaccessibility of such compounds was 57.4 (white tea) to 73.2% (green tea). Therefore, the technique used presents a viable alternative for the protection of bioactive compounds and studies can be carried out for the application of the biosorbed material in foods.

In addition to the concern with the preservation of bioactive compounds during gastrointestinal digestion, agricultural residues that may be present in the leaves of *C. sinensis* have been investigated. Inevitably, the infusion process collaborates with the extraction of undesirable compounds such as imidacloprid and acetamiprid, from pesticides used in plant cultivation. The use of activated carbon, an effective and low-cost adsorbent, presents removal rates of these compounds above 85% (Lu et al., 2022). However, the non-selective adsorbent used has wide applicability, especially in case of poisoning by chemical substances and removal of impurities and may also interfere with the concentration of compounds of interest. The process used in this study did not interfere with the amount of free amino acids; however, some phenolic compounds such as catechin, CG, GC, GCG, epicatechin, EGC and EGCG showed a reduction of up to 30%. The concentration of total phenolic compounds remaining in the tea was above 85%, meeting the minimum concentration of 500 mg/kg required by the Chinese National Standard (Lu et al., 2022).

In the last decade, there has been a great advance in the understanding of the mechanisms involved in the bioaccessibility of different food matrices, especially teas. However, it is common sense that the bioactive compounds in teas are not absorbed in the same way and several factors can interfere with bioaccessibility. Therefore, new production and processing methods are necessary to ensure a better stability and bioavailability of teas.

As an example of these methods, it can be cited the use of unconventional solvents (Sanz, Flórez-Fernández, Domínguez, & Torres, 2020), negative pressure extraction (Roohinejad et al., 2016, Sanz et al., 2020), supercritical extraction with CO_2_ (Bermejo et al., 2016, Sanz et al., 2020), pressurized liquid extraction (Sanz et al., 2020), extraction with subcritical water (Wu et al., 2018), ultrasound-assisted extraction (Sáenz-Galindo et al., 2021), and extraction by filter membranes (Sanz et al., 2020).

Other industrial applications of teas and their bioactive compounds have been explored. Innovation food technology have been the main targets of research. The following topic presents the main applications of teas or bioactive compounds extracted from teas in food products.

## Applications of phenolic compounds of teas in food products

As previously mentioned, the main antioxidants in teas (*C. sinensis*) comprise those belonging to the flavonoid family and the flavan-3-ols subgroup, known as catechins, epicatechins and their derivatives. These compounds have important biological actions, both for human health and for the shelf life of food products, and have been standing out in different industrial segments (Fan et al., 2021, Pedro et al., 2019).

*In vitro* and *in vivo* studies show that tea intake (*C. sinensis*) is associated with biological activities in the human body, through the effects of decreasing reactive oxygen species (ROS) and oxidative damage to DNA (Kapoor, Sugita, Nishimura, Sudo, & Okubo, 2018). Biological effects are associated with reducing the risk of developing and treating cancerous tumors (Bhattacharya et al., 2020, Haghparasti and Mahdavi Shahri, 2018, Rawangkan et al., 2018, Shin et al., 2018) attenuating effect on inflammatory processes through signaling pathways (Fechtner et al., 2017, Kim et al., 2020, Su et al., 2019), protection against cardiovascular disease (Chung et al., 2020), hepatoprotective actions (Cao et al., 2020, Stefan et al., 2019), prevention and treatment of diabetes mellitus (Cheng et al., 2020, Meng et al., 2019), neuroprotection and immune modulation (Schimidt et al., 2018, Sharma et al., 2017), in addition to having recently filed a response against SARS-Cov-2 (Zhu & Xie, 2020).

**Fig. 4:**
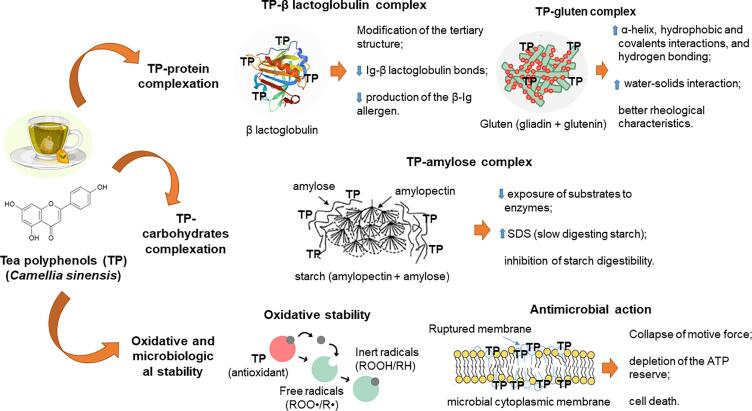
Mechanisms of action of tea polyphenols (TP) in food products.

The use and application of bioactive compounds from *Camellia sinensis* teas by the food industry is promising. Natural antioxidants can be used as additives, as they act with different functions in foods through interaction/molecular modification mechanisms (Fig. 4), enabling the development of innovative, healthy, nutritious and long shelf-life products (Albuquerque, Oliveira, Barros, & Ferreira, 2020).

The interaction of phenolic compounds-proteins and phenolic-compounds-carbohydrates is an important area of research for the modification of macromolecules and for food processing. The interaction of *C. sinensis* phenolic compounds and proteins is mentioned in the literature as an innovative strategy used in the formulation of hypoallergenic dairy products and bakery products with better rheological properties. Furthermore, the polyphenol-carbohydrate complex is a potential ingredient for the development of dietary products.

Pessato et al. (2018) complexed (−)-epigallocatechin gallate (EGCG) derived from green tea to whey protein isolate under acidic and neutral pH conditions at 25 °C. The EGCG molecule has gallyl, galloyl and hydroxyl groups, which contributed to the effective complexation with proteins. The results showed a reduction in the most common allergen components found in milk, β-Ig and BSA. These allergens are resistant to pepsin in the acidic environment of the stomach and are absorbed intact. EGCG-protein complexation causes modification of the tertiary structure of the protein, reducing IgE binding to β-lactoglobulin and, consequently, decreasing the production of the β-Ig allergen.

Green tea extracts are cited as improvers of the physicochemical and rheological properties of noodles. Xu, Wu, Hou, and Du (2019) showed that incorporating 1–2% green tea extract into traditional Chinese noodles improved dough strength and promoted starch gelatinization. In addition, the product developed showed a decrease in the parameters of hardness, chewability and glycemic index, in addition to an increase in antioxidant capacity and digestibility, when compared to the control sample (without addition of extract). Han, Ma, Zhang, Li, and Sun (2020) also demonstrated that the noodles enriched with phenolic compounds from green tea showed increased antioxidant activity and a more continuous and ordered gluten network, with better quality of traction, texture and chewing parameters. The phenolic compounds in green tea promote molecular changes between gluten proteins (gliadin and glutenin), such as the increase in the α-helix, hydrophobic interactions, covalents and bonds by hydrogen bonds. Furthermore, these compounds induce the exchange between SH/SS bonds and the polymerization of the molecular chains of gluten proteins, causing an increase in the height and width of the chains and in the water-solids interaction. These factors play a fundamental role in the formation of a three-dimensional viscoelastic gluten network with better rheological characteristics.

Studies demonstrate that tea polyphenols (TP) can adjust glucose release from the liver, activate insulin receptors, and inhibit carbohydrate digestion and absorption (Cheng et al., 2020, Meng et al., 2019). These characteristics are useful for the use of tea polyphenols in the development of dietary products, as shown in the study by Lv et al. (2019). The study showed that the incorporation of TP through potato starch milling changed the proportions of RDS (fast digesting starch), SDS (slow digesting starch) and RS (resistant starch). The obtaining of a high proportion of SDS indicated that the TP-starch complexation can be useful for the development of products aimed at postprandial glycemic control. Milling causes destruction of the crystalline structure of starch granules (amylose and amylopectin), increasing the exposure of hydroxyl groups and complexing TP-amylose. Complexation leads to less exposure of substrates to enzymes and causes starch to have a slow digestion property. Inhibition of starch digestibility is due to hydrogen bonding between tea polyphenol hydroxyl groups and digestive enzymes (dose-dependent).

In addition to technological purposes, researchers suggest that tea polyphenols (TP) contribute to increasing the shelf life of food products, through the inhibition of microorganisms and oxidative processes. In addition, the polyphenols from tea have been widely used to replace synthetic antioxidants. Studies demonstrate that EGCG from teas promote antimicrobial action through the disruption of the microbial cell membrane. The hydroxyls of the EGCG molecule cause delocalization of electrons, which act as proton exchangers, reducing the gradient across the cytoplasmic membrane. This will lead to breakdown of motive power, depletion of the ATP reserve and cell death. In addition to the antimicrobial effect, EGCG act as inhibitors of oxidative chain reactions, through the donation of an active hydrogen atom to free radicals (ROO• and R•) (Schilling et al., 2018).

Souza et al. (2020) incorporated flavonoids extracted from black tea in the formulation of bakery products. The incorporation of bioactive compounds in breads enhanced color and prolonged shelf life, through inhibition of lipid oxidation and antimicrobial action. Furthermore, the flavonoids showed stability to thermal processing (∼180 °C). The study by Thakaeng, Wongsakul, and Mat Yusoff (2020) showed that the addition of 6% (w/w) of green tea extract in the butter formulation inhibited lipid oxidation and microbiological growth, without compromising the sensory acceptance of the product. Gramza-Michałowska, Kulczyński, Skopiec, Kobus-Cisowska, and Brzozowska (2021) applied the powdered extract of yellow tea in the formulation of white, milk and bitter chocolates. The addition of 2% of yellow tea extract in the chocolate formulation provided an increase in sensory acceptance, antioxidant activity and inhibition of lipid oxidation by 50, 42 and 12% for milk, dark and white chocolates, respectively.

Meat products contain high concentrations of proteins and lipids and are susceptible to degradation by oxidative and microbiological processes. Tea extracts have been cited as efficient preservatives for meat products. Bellés, Alonso, Roncalés, and Beltrán (2017) showed that spraying 0.5% (w/v) green tea extract onto lamb chops increased shelf life from 8 to 11 days. Inhibition of lipid oxidation resulted in the maintenance of the red color of the meat and less formation of metmyoglobin. However, 5% concentrations of the extract resulted in unacceptable color changes. Purnamayanti, Jamhari, Hanim, and Irawan (2020) also studied the shelf life of lamb meat and showed that the incorporation of green tea extract in lamb sausage inhibited bacterial growth and lipid oxidation. With the same purpose, Jayawardana, Warnasooriya, Thotawattage, Dharmasena, and Liyanage (2019) showed that the incorporation of 0.05 and 0.30% of green and black tea extract, respectively, in the formulation of uncured pork sausages provided inhibition of lipid peroxidation (reduction of TBARS - thiobarbituric acid reactive substances) without changing the color, odor, texture, juiciness or general acceptance. Kırmızıkaya, Karakaya, and Babaoğlu (2021) tested the application of powders and infusions of black, green, and white teas in minced meat and analyzed the lipid oxidation during 7 days of storage under refrigeration. All tea samples showed inhibition of lipid oxidation in minced meat, however, as mentioned by other authors, high concentrations have low sensory scores compared to control samples. Therefore, the incorporation of different concentrations of tea extracts in meat products must be carefully investigated to obtain desirable antioxidant and sensory properties.

Teas have been used for centuries by humanity. The important sensory, nutritional, chemical, phytochemical, and biological properties of teas make these infusions promising for technological application in the formulation of food products. Whether in the form of crude extract or in the form of isolated individual compounds, *Camellia sinensis* teas have the most diverse applications in the preservation of meat products, in improving the sensory and rheological properties of bakery products, in the replacement of synthetic antioxidants, in the reduction of allergy in dairy products, among others.

## Conclusion

Tea production from *C. sinensis* is a billion-dollar industry. The consumption of teas goes beyond a pleasant and cultural activity. Teas are major sources of bioactive compounds that may bring health benefits, mainly because their hypoglycemic, antioxidant, anti-inflammatory, neuroprotective, anticarcinogenic, anti-obesity, cardiovascular and liver protection properties. Processing and gastrointestinal digestion of C. sinensis teas influences their chemical composition and upcoming biological properties. Flavan-3-ols, such as catechins and epicatechins, the main phenolic compounds in teas, are highly unstable to processing conditions and gastrointestinal digestion. Thus, new technologies for processing and preserving the phenolic compounds of C. sinensis have been evaluated, especially to increase their bioaccessibility, such as encapsulation and biosorption in Saccharomyces cerevisiae yeast cells. Research on teas have grown substantially over the years and the main topics investigated are related to their bioactive profile and the use of extracts or isolated compounds to develop new products for food and pharmaceutical industries. Food products produced with C. sinensis show increased shelf life (meat products) and improved mechanical and sensory properties (bakery and dairy products). Hence, tea extracts are promising ingredients for food industry applications.

## Declaration of Competing Interest

The authors declare that they have no known competing financial interests or personal relationships that could have appeared to influence the work reported in this paper.
